# Tailoring spontaneous infrared emission of HgTe quantum dots with laser-printed plasmonic arrays

**DOI:** 10.1038/s41377-020-0247-6

**Published:** 2020-02-04

**Authors:** A. A. Sergeev, D. V. Pavlov, A. A. Kuchmizhak, M. V. Lapine, W. K. Yiu, Y. Dong, N. Ke, S. Juodkazis, N. Zhao, S. V. Kershaw, A. L. Rogach

**Affiliations:** 1 Institute of Automation and Control Processes, Far Eastern Branch, Russian Academy of Sciences, Vladivostok, 690041 Russia; 2 Far Eastern Federal University, Vladivostok, 690090 Russia; 3 University of Technology Sydney, NSW 2007 Sydney, Australia; 4 Department of Materials Science and Engineering, and Centre for Functional Photonics (CFP), City University of Hong Kong, Kowloon, Hong Kong SAR China; 5 Engineering Research Center of Nano-Geomaterials of Ministry of Education, Faculty of Material Science and Chemistry, China University of Geosciences, Wuhan, 430074 China; 6 Department of Electronic Engineering, The Chinese University of Hong Kong, Shatin, New Territories, Hong Kong SAR China; 7 Swinburne University of Technology, John St., Hawthorn, VIC 3122 Australia; 8 Melbourne Centre for Nanofabrication, ANFF, 151 Wellington Road, Clayton, VIC 3168 Australia

**Keywords:** Optics and photonics, Optical materials and structures

## Abstract

Chemically synthesized near-infrared to mid-infrared (IR) colloidal quantum dots (QDs) offer a promising platform for the realization of devices including emitters, detectors, security, and sensor systems. However, at longer wavelengths, the quantum yield of such QDs decreases as the radiative emission rate drops following Fermi’s golden rule, while non-radiative recombination channels compete with light emission. Control over the radiative and non-radiative channels of the IR-emitting QDs is crucially important to improve the performance of IR-range devices. Here, we demonstrate strong enhancement of the spontaneous emission rate of near- to mid-IR HgTe QDs coupled to periodically arranged plasmonic nanoantennas, in the form of nanobumps, produced on the surface of glass-supported Au films via ablation-free direct femtosecond laser printing. The enhancement is achieved by simultaneous radiative coupling of the emission that spectrally matches the first-order lattice resonance of the arrays, as well as more efficient photoluminescence excitation provided by coupling of the pump radiation to the local surface plasmon resonances of the isolated nanoantennas. Moreover, coupling of the HgTe QDs to the lattice plasmons reduces the influence of non-radiative decay losses mediated by the formation of polarons formed between QD surface-trapped carriers and the IR absorption bands of dodecanethiol used as a ligand on the QDs, allowing us to improve the shape of the emission spectrum through a reduction in the spectral dip related to this ligand coupling. Considering the ease of the chemical synthesis and processing of the HgTe QDs combined with the scalability of the direct laser fabrication of nanoantennas with tailored plasmonic responses, our results provide an important step towards the design of IR-range devices for various applications.

## Quantum dots: A boost for new sensing technologies

By boosting the emission of semiconductor quantum dots, scientists have laid the foundations for new technologies for a range of applications, including night vision and security systems, sensors and spectroscopy tools. Colloidal semiconductor quantum dots (QDs) with high photoluminescence quantum yield (PLQY) in the near-to-mid infrared range are a promising new material for use in sensing technologies. However, at longer wavelengths, the quantum yield drops, limiting their applications. An international team of researchers, led by Andrey Rogach and colleagues from the City University of Hong Kong, has now achieved a five-fold enhancement in the PLQY of mercury telluride QDs. By coupling the QDs to a lattice of plasmonic nanoantennas arranged on gold films, they were able to control the radiative and non-radiative channels of the QDs. The work is an important step towards infrared-range devices.

## Introduction

Inexpensive near-infrared (IR) to mid-IR sources and detectors operating at room temperature are expected to revolutionize current technologies for the realization of various night vision and security systems, sensing and spectroscopy tools, etc. Colloidal semiconductor quantum dots (QDs) characterized by a high photoluminescence quantum yield (PLQY), which currently reaches at least 40% for QDs in the 1–2 μm range, represent a promising material for the realization of these devices^1–3^. However, at longer wavelengths, the QY rapidly drops as the radiative emission rate decreases following Fermi’s golden rule, and non-radiative recombination channels compete more effectively with light emission. Control over the emission properties of the QDs is expected to provide a way to achieve the ultimate performance of such devices, providing a means of restoring the chances of radiative emission over non-radiative recombination. The emission properties of any quantum emitter are known to be strongly modified in the vicinity of a plasmonic nanostructure, which can resonantly interact with pump radiation via excitation of collective oscillations of a free electron plasma^4^. In particular, both spontaneous radiative and non-radiative emission rates, the lifetime of an excited state, the directionality and the emission spectrum can be significantly tailored^5–11^, as predicted in the pioneering work of Purcell^12^. Ordered arrays of plasmonic nanoantennas allow for the realization of low-loss collective resonances via the excitation of specific diffractively coupled lattice modes^13–20^. Such arrays are typically up-scalable to square millimeter areas, which opens a pathway for realistic applications and devices based upon them. To achieve the best performance with such an approach, both the absorption and emission bands of the emitter should simultaneously fit the plasmonic resonances of the nanostructures. Moreover, real-life applications will require millions of high-quality plasmonic nanoantennas arranged into well-ordered arrays, which makes their fabrication with existing lithographic techniques time-consuming and cost-consuming.

One factor that governs the limiting IR emission wavelength of QDs is the bandgap position of the bulk material since quantum confinement introduces a blueshift of this value. Whilst lead chalcogenides have bandgaps of 0.37–0.26 eV, materials such as HgSe (−0.06 eV), HgTe (−0.15 eV) and Sn (−0.413 eV) have no bandgap in the bulk, and they are semi-metals rather than semiconductors. Under these circumstances, at some particle size going from the bulk towards small particle dimensions, the effect of quantum confinement introduces a transition from semi-metal to semiconductor behavior, and the latter would in principle have a true zero bandgap just at this transition. Consequently, these zero bandgap materials could offer the widest IR interband transition ranges of all the QDs. In practice, so far, the longest wavelength for HgTe QD bandgaps has been observed by the Guyot-Sionnest group, with weak interband emission peaking at approximately 8300 nm and an absorption edge at approximately 6600 nm. However, since the particle size distributions are relatively broad and the tail of the absorption spectrum extends to just over 1000 cm^−1^, IR photocurrent spectra were recorded out to 12 µm^21^. By the time such long wavelengths are attained, the PLQY is extremely small (≪0.01%)^22,23^. In the present work, by comparison, the HgTe QDs used were not very large, and their emission was stronger than that for the longer wavelength-emitting examples just cited. From the strength of the PL emission, we would estimate that the QYs of these QDs are in the same range as those in our previous paper^22^; i.e., at the wavelengths concerned herein, the PLQYs are estimated to range from 1 to 10%, decreasing as the emission wavelength and particle size increase.

Thus far, the main interest in QDs at wavelengths beyond 2000 nm stems from the size tuning of the absorption edge and its exploitation in IR photodetectors^24–35^; moreover, there is a strong interest in increasing the PLQY values to possibly open up emissive applications to longer wavelengths than those at present as well as to improve photodetector operation. To this end, several groups, ourselves included, have attempted to make HgTe-based core/shell QDs, but improvements to the PLQY were rather limited, though the shells did improve the thermal stability of the dots, especially in film form^36,37^. Notwithstanding the use of heterostructures, at long wavelengths (e.g., >5000 nm), the PLQY at best remains <0.1%. The benefit of using core/shells did not amount to the sort of PLQY improvement often routinely seen for shorter wavelength, visible-range-emitting QDs (see, for example, numerous cases cited in our recent review by Jing et al.^38^).

Another key concern when trying to maintain QD PLQY when shifting bandgaps further into the IR is that of trying to eliminate or greatly suppress a major non-radiative recombination channel via coupling to IR overtone and combination absorption bands in the surrounding medium^1,23,39^. This issue has been addressed by replacing the surrounding medium with IR-transparent materials such as As_2_S_3_^26,40^. Similar coupling to nearby overtone and combination bands has been considered with other IR range nanomaterials such as carbon nanotubes as well as QDs by Velizhanin^41^. Whilst some improvements were obtained using As_2_S_3_ hosts and shells^26,40^, the overall performance benefits were not very large, and a substantial scope for greater enhancements still remains. Other recent work on IR QDs, and Hg chalcogenide QDs in particular, has focused on avoiding the necessity of growing such large particles by exploiting comparatively lower energy intraband transitions at a given QD diameter^32,42,43^.

Whether IR QDs are to be used as emitters or as the absorbing medium in a photodetector, their performance in both types of application can benefit from them having a more intense PL^29^. At a rather simplistic level, both emission and photocarrier extraction are improved if other competing non-radiative recombination processes are slower by comparison. Our approach in the present work of combining HgTe QDs with photonic nanostructures to bring about such recombination rate manipulation is not restricted to such QDs alone and could equally well be used with Pb chalcogenide or other types of IR QDs; our choice of QD simply arose from our previous experience in the synthesis of HgTe QDs and their ready availability in our labs.

Though the preceding background on the IR QD state of the art is by no means a comprehensive review of the field, it may be appreciated that whilst colloidal QDs can offer desirable features such as simple wet chemical synthesis and film deposition methods (spin coating, dip coating, spray coating^44^, or even QD inks^31^), along with a simple means to control the bandgap position through size regulation, there are also often other aspects of their photophysics that can provide technical barriers to their use in many optoelectronic applications. Combining QDs with other interesting nano-technologies and micro-technologies—such as plasmonic particles^35^, photonic nanostructures^28^, or plasmonic nanostructures as used herein—is a useful method to capitalize on the positive benefits of QD engineering whilst circumventing some of their current weaknesses or gaining synergistic benefits from the combination of QDs with other technologies.

**Fig. 1:**
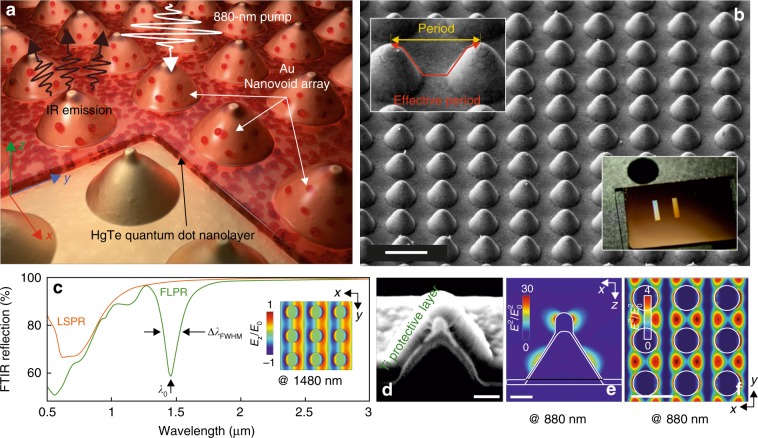
Laser-printed plasmonic nanobump array for enhancement of the spontaneous emission rate of IR-emitting QDs. **a** Artistic representation of the HgTe QD layer coated above the laser-printed Au nanobump array. **b** Side-view (view angle of 45°) SEM image showing the Au nanobump array printed at a 1-μm pitch (scale bar corresponds to 1 µm). A close-up SEM image on the top inset demonstrates the difference between the period and the “effective” period of the nanobump array. The bottom inset shows a photograph of two large-scale (3 × 9 mm^2^) nanobump arrays produced on the glass-supported Au film. **c** Typical Fourier transform infrared (FTIR) reflection spectrum of the plasmonic nanobump array printed at a 1 µm pitch (green curve). The contribution of the localized surface plasmon resonance (LSPR) of the isolated nanobumps of a given shape is shown by the orange dashed curve. FLPR denotes the first-order lattice plasmon resonance. The inset provides the distribution of the z-component of the EM field (*E*_z_/*E*_0_) calculated 50 nm above the smooth Au film surface at 1480 nm wavelength. Circles indicate the nanobump positions. The details related to the calculations of the LSPRs and FLPRs are provided in the Supporting Information. **d** Side-view (view angle of 70°) SEM image of the cross-section of the nanobump (scale bar is 200 nm). **e**, **f** Calculated EM-field intensity distribution (*E*^2^/*E*_0_^2^) near the isolated nanobump (in the *xz* plane) and 50 nm above the smooth Au film level (in the *xy* plane) at an 880 nm pump wavelength (scale bars in **e**, **f** are 200, 1000 nm, respectively)

In this paper, we demonstrate an efficient tuning of the spontaneous emission of near- to mid-IR-emitting HgTe QDs placed above a high-quality plasmonic nanobump array produced on the surface of glass-supported Au films via inexpensive ablation-free direct femtosecond (fs) laser patterning (see Fig. 1a). By taking advantage of the facile tuning of the spectral position of the lattice and localized surface plasmon resonances (LSPRs) originating from both the laser-printed nanobump geometry and arrangement, we achieved a five-fold enhancement of the emission intensity of the HgTe QDs. This enhancement was attributed to radiative coupling of the spontaneous emission spectrally matching the first-order lattice resonance of the nanobump array as well as more efficient PL excitation provided by coupling of the pump radiation to the LSPR of the isolated structures. Moreover, matching the first-order lattice resonance of the nanobump array with a vibration band of dodecanethiol (DDT) ligands of HgTe QDs was shown to reduce the influence of the non-radiative PL decay versus radiative recombination, allowing us to improve the shape of their emission spectrum through a reduction in the spectral dip related to ligand coupling.

## Results

### Fabrication of laser-printed Au nanobump arrays with tailored plasmonic response

Plasmonic arrays composed of ordered nanobumps were fabricated via direct fs-laser patterning of a 50-nm thick glass-supported Au film (Fig. 1b) according to the procedure described in the Materials and methods section. As previously shown, tightly focused fs-laser pulses with an incident fluence ranging from ≈70 to 130 mJ/cm^2^ (at 515 nm incident wavelength) are able to cause delicate local melting of the metal film, which acoustically relaxes from the supporting substrate and resolidifies in the form of hollow nanobumps^45,46^ (see top inset in Fig. 1b). For the fixed optical size of the laser spot, the characteristic shape and size of the produced structures are defined by the incident pulse fluence. Importantly, such a procedure provides debris-free patterning, ensuring remarkably high reproducibility of the nanobump shape even over large mm-scale areas. The fabricated nanobump lattices had a size of 3 × 9 mm^2^ and contained approximately 10^8^ individual nanobumps arranged into rectangular-shaped arrays at variable pitch dimension *p* for each array (see bottom inset in Fig. 1b).

As previously shown^45^, irradiation of near-IR light at normal incidence onto such arrays can be efficiently coupled to surface plasmon polariton (SPP) waves. The SPPs are resonantly enhanced by the array, producing a fairly narrow resonance (further referred to as a first-order lattice plasmon resonance, FLPR) with a quality factor defined as a full-width half-maximum value divided by a resonant wavelength *λ*_0_/*λ*_FWHM_ ≈ 13, which can be revealed in the Fourier transform infrared (FTIR) reflection spectrum (green dashed curve, Fig. 1c). More specifically, the spectral position of the FLPR (as well as similar higher-order lattice resonances; see inset in Fig. 1c) is defined by an “effective” period representing the distance that an excited SPP travels along the slopes of a nanobump plus the remaining distance along the metal film (see top inset in Fig. 1b).

The spectral position of these resonances as well as their amplitude can be easily tailored by adjusting both the actual lattice pitch *p* and the geometric parameters of the individual nanobumps (see Fig. S1 in Supporting Information). The spectral position of the first-order and second-order lattice resonances as a function of the “effective” array period is given in Fig. S2 of the Supporting Information. At the same time, isolated nanobumps also support multiple LSPRs (orange dashed curve in Fig. 1c and curve in Fig. S3 in Supporting Information), which are expected to contribute to the enhancement of electromagnetic near-fields around the nanostructures in the visible and near-IR parts of the spectrum, in accordance with our previous studies^47,48^. Along with the multi-order lattice resonances, these LSPRs appear as a broad near-IR shoulder seen in the FTIR reflection spectrum (Fig. 1c); they can easily be visualized by measuring the dark-field back-scattered spectrum in the mentioned spectral region. To reveal the build-up of the electromagnetic near-fields mediated by the LSPR at the PL pump wavelength (880 nm), we performed 3D finite-difference time-domain (3D FDTD) simulations (see Materials and methods section). The normalized squared electric field amplitude *E*^2^/*E*^2^_0_ calculated near the isolated nanobumps is shown in Fig. 1e. A substantial enhancement of the *E*^2^/*E*^2^_0_ value by as much as 30-fold can be found near the very tip of the conical-shaped nanobump as well as near its sidewalls; these two groups of electromagnetic “hot spots” are attributed to the LSPR of the nanobump tip and the running plasmon wave partially bounded within the curved contour of the nanobump walls^47^, respectively. Moreover, constructive interference of the launched SPPs produces locally enhanced EM fields, which cover a considerable part of smooth Au film sections between the nanobumps, as revealed by similar numerical calculations (see Fig. 1f). With such dependence of the various plasmonic resonances on the geometry of the nanobumps and the array period, it is possible to adjust the geometry of the overall array structure at the fabrication stage to simultaneously match the LSPR of the isolated nanostructure to the absorption band and the main resonance related to the effective period to match the emission band for a given quantum emitter.

### Tailoring the spontaneous emission of HgTe QDs coupled to the plasmonic array

**Fig. 2:**
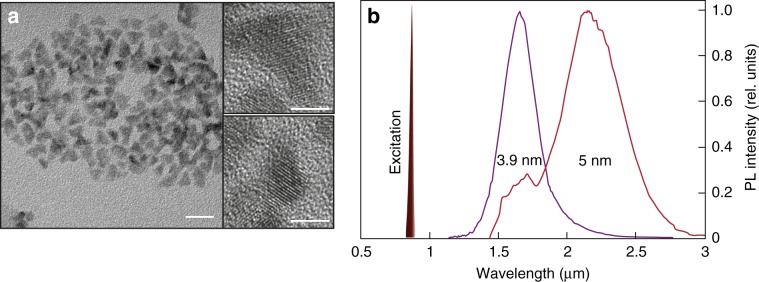
IR-emitting HgTe QDs. **a** TEM images of the HgTe QDs used in this study (scale bar is 20 nm); insets show a magnified view of selected QDs (scale bars are 5 nm). **b** Emission spectra of HgTe QDs with average sizes of 3.9 and 5.0 nm. Also shown is the spectral position of the PL excitation at 880 nm

The HgTe QDs used in this study were synthesized using a gaseous H_2_Te precursor via an aprotic solvent method described in the Materials and methods section. Transmission electron microscopy (TEM) images of HgTe QDs are presented in Fig. 2a. The pyramid-shaped particles typically show randomly truncated corners. We utilized two types of HgTe QDs with characteristic average sizes of 3.9 and 5.0 nm with emission maxima centered at 1.6 and 2.2 μm, respectively (Fig. 2b). The 3.9 nm QD sample has a reasonably sharp absorption edge and a narrower emission peak than that of the 5.0 nm sample, where the emission is broader and the absorption edge appears less sharp, indicating a broader size dispersion in the latter case. The 5.0 nm sample also shows a spectral dip of approximately 1650 nm, which is due to coupling via a polaron mechanism involving trapped carriers coupled to overtone and combination bands in the DDT ligand used to stabilize the QDs^1^. This dip is absent in the 3.9 nm material, which has a higher quality and reduced carrier trapping. Upon deposition as thin solid films, there was a slight (few nm) redshift in the PL maxima for both samples.

**Fig. 3:**
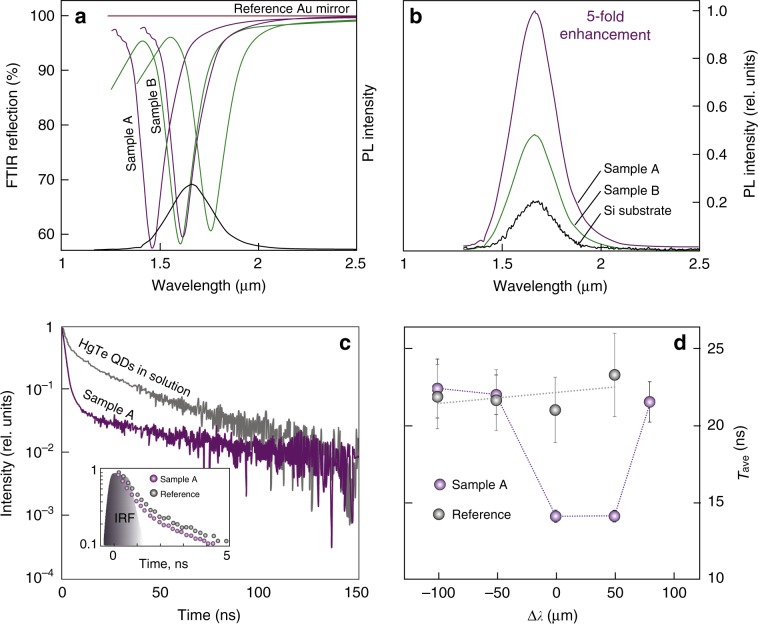
Emission enhancement of HgTe QDs deposited on a laser-printed plasmonic array. **a** FTIR reflection spectra of two plasmonic nanobump arrays printed at pitches of 1 and 1.1 μm (Samples A and B, respectively; solid lines—as-fabricated arrays; dashed lines—arrays after HgTe QD deposition). All spectra are normalized to the reflection spectrum of a smooth Au film, shown as a red line. The PL spectrum of the 3.9 nm HgTe QDs in solution is shown as a black line. **b** Emission spectra of the HgTe QD layer deposited on a crystalline Si substrate (black), Sample A (purple), and Sample B (green). Spectra obtained for each sample are averaged over 20 similar measurements from different sample areas and normalized over the maximal PL intensity value. **c** The PL decay of the HgTe QDs in solution (gray) and on Sample A (purple). The inset provides a magnified view of the initial part of the decays to better illustrate the difference between the decay curves of the HgTe QDs deposited on a Au/Si reference sample and Sample A; the filled area denotes the measured instrument response function (IRF). **d** Average (*τ*_ave_) PL decay times of HgTe QDs deposited on a Si reference substrate and on Sample A at various emission wavelengths spectrally shifted by Δ*λ* with respect to the first-order lattice resonance

The nanobump arrays were covered with a layer of QDs with an overall AFM-verified thickness of ≈90 nm. Emission of the QDs was excited with linearly polarized normal-incident 880-nm wavelength laser radiation shaped into a 300-μm diameter spot using a focusing lens (see Materials and methods section). Different parts of the rather large (3 × 9 mm^2^) samples were probed to provide statistical averaging of the results. We tested two plasmonic arrays with close first-order lattice resonances centered at 1480 ± 30 and 1600 ± 30 nm (further referred to as Sample A and Sample B, respectively). Both arrays have a lateral size of 3 × 9 mm^2^ and comprise identical conical-shaped nanobumps arranged into rectangular-shaped lattices at slightly different pitches of 1 and 1.1 μm, providing a scalable redshift of the FLPR^45,49^. Notably, the PL maximum of the HgTe QDs coincided well with the spectral position of the first-order lattice resonance of the as-fabricated Sample B, while the same plasmonic band of Sample A was blueshifted with respect to the QD emission maximum (solid curves in Fig. 3a). Surprisingly, Sample A provided at least five-fold more enhanced PL intensity from the HgTe QDs compared to that of the reference signal measured from the same layer covering either a c-Si slide or a smooth Au film, in contrast to Sample B, which provided only a 2.5-fold average enhancement (Fig. 3b). Aside from the unexpected difference between the PL intensity obtained for the two plasmonic arrays, which will be discussed later, both samples provided detectable enhancement of the spontaneous emission from the HgTe QD layer when compared to that from the reference Si or smooth Au substrates. This enhancement cannot be explained by the slightly enlarged (≈1.3-fold) surface area of the plasmonic array compared to that of the smooth reference and appears to be governed by the cumulative action of several factors.

First, compared to HgTe QDs in solution, QDs deposited on both kinds of plasmonic arrays provided a faster radiative emission rate via a reduced PL decay lifetime (Fig. 3c). The obtained PL decay curves were fitted with a triexponential model described by the equation *F*(*t*) = *A*_s_exp(−*t*/*τ*_s_) + *A*_1_exp(−*t*/*τ*_1_) + *A*_2_exp(−*t*/*τ*_2_), where *τ*_1_ and *τ*_2_ represent the decay lifetimes, which correspond to either fast or slow components having amplitudes of *A*_1_ and *A*_2_, respectively. The fast component indicates excitation energy migration between close-packed dots (e.g., via a FRET mechanism) as well as trapping of the exciton by a non-luminescent site, such as a QD defect or its blinking “off-state” ^50,51^. The slow component corresponds to PL decay from isolated QDs. In turn, *τ*_s_ and *A*_s_ are the time constant and amplitude, respectively, which are associated with the instrumental response function (IRF) to the scattered pump radiation passing through the emission monochromator at the emission band and have to be accounted for due to the relatively low absolute PL intensity of the deposited QDs (see Inset on Fig. 3c). In our studies, we detected an increase in the decay rate of both fast and slow components for the QDs on the plasmonic structure. Thus, we used the average decay value to describe this process. The average PL lifetime (*τ*_ave_) was calculated via the weighted averaging of the lifetime components by the amplitudes. Analysis of the obtained data indicated a faster emission rate (*τ*_ave_ = 14.1 ± 0.4 ns) for the HgTe QDs deposited on the plasmonic array than that for the QDs placed over a smooth Au (or Si) reference (*τ*_ave_ = 21 ± 2 ns). More detailed studies of the PL decays for Sample A probed at various emission wavelengths revealed the direct correlation between the decrease in the average decay lifetime and the detuning of the probing wavelength with respect to the first-order lattice resonance (see Fig. 3d).

Second, when using an 880-nm pump to excite the spontaneous emission of the HgTe QDs, the array of conical-shaped nanobumps produces enhanced electromagnetic fields mediated by the LSPRs and provides constructive interference of the SPPs, as revealed by corresponding FDTD calculations (see Materials and methods section). These numerical calculations give an substantial enhancement of the near-field intensity *E*^2^/*E*_0_^2^ by as much as 30-fold at the vicinity of the very tip and the sidewalls of the conical-shaped nanovoid as well as an average 4-fold increase in *E*^2^/*E*_0_^2^ near the smooth metal film between the nanobumps (see Fig. 1e, f). In this way, a considerable amount of the QDs placed near these electromagnetic “hot spots” will be pumped more efficiently, resulting in higher PL intensity.

The precise spectral matching of the PL band of the HgTe QDs with the first-order lattice resonance also affected the radiative part of the spontaneous emission. In particular, for almost identical excitation conditions realized for Samples A and B, the average PL intensity of the HgTe QD layer differs by a factor of 2 (Fig. 3b). This effect can be attributed to efficient outcoupling and improved directionality of the emitted photons by the plasmonic grating in the direction perpendicular to its surface. We also observed a weaker PL intensity for the plasmonic array (Sample B, Fig. 3a, b) initially providing better spectral matching of the first-order lattice resonance with the PL emission. This observation, however, could be reconciled by taking into account the plasmonic nature of the lattice resonance. The deposited HgTe QD layer causes a change in the local effective refractive index of the SPP wave at the (previously) air–metal interface, resulting in a substantial spectral redshift of the plasmonic lattice resonances (dashed curves in Fig. 3a), which causes a fortuitously improved spectral matching of the main band of Sample A with the PL maximum of the HgTe QDs. As the exact value of the refractive index of such a layer is difficult to measure directly, we performed a series of calibration experiments by applying thin dielectric films of known refractive index and variable thickness *d* above the nanobump arrays to quantitatively assess the spectral shift of the lattice band with regard to the variation in the local effective refractive index. Within the range of available materials for such a procedure, we chose amorphous silicon (α-Si) coated above several similar plasmonic nanobump arrays using a commercial *e*-beam evaporation system equipped with a calibrated quartz microbalance. This material has an average bulk refractive index of *n*_α−Si_ ≈ 3.47 in the near-IR spectral range^52^, while the bulk refractive index of HgTe is close to this value according to the available experimental data^53^. The relative FLPR spectral shift ∆*λ*/*λ*_0_ versus the thickness *d* of α-Si dielectric films is shown in Fig. S4 of the Supporting Information, revealing competitive sensitivity of this resonance to even very thin dielectric layers coated on top of the plasmonic array. With the spectral resolution of our FTIR setup (≈1 nm) in this wavelength range, it is possible to identify the presence of a few-nanometer-thick high-index dielectric layer coated above the structures that provides a feedback signal for assessment of its actual thickness. Based on such an assessment, the thickness of the HgTe QDs deposited on Samples A and B appeared to be ≈90 nm, which correlates well with the thickness of the HgTe QD layer determined by AFM.

**Fig. 4:**
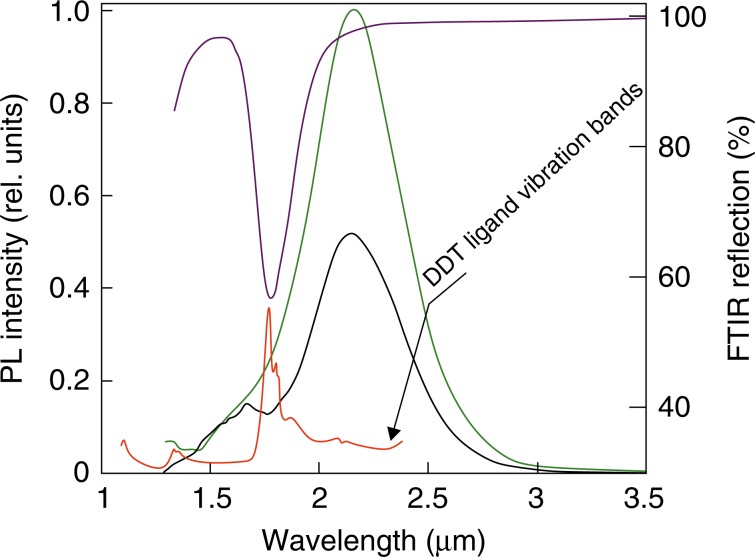
Tailoring the IR emission spectra of the HgTe QD film with a laser-printed plasmonic array. Emission spectrum of the 5.0-nm size HgTe QD layer deposited on a reference Si substrate (black) and a plasmonic nanobump array (green). Also shown are a normalized FTIR reflection spectrum of the corresponding plasmonic nanobump arrays coated with the QD layer (purple) as well as part of the near-IR absorption spectrum of the DDT ligand of the HgTe QDs (red)

Finally, we found that the coupling of the HgTe QDs to the plasmonic array allows for enhancement of their mid-IR spontaneous emission spectrum in some cases. In particular, IR vibration bands of the organic DDT ligand used to stabilize the QDs provide an efficient non-radiative decay channel resulting in a considerable spectral dip, which can be seen, for example, in the spontaneous emission spectrum of the larger 5.0-nm size HgTe QDs emitting at 2.23 μm (red curve in Fig. 4). In a previous study^1^, we identified that such ligand vibration overtone and combination bands can couple directly with QD excitons, either through an electronic-to-vibrational Forster-type resonant energy transfer channel or in QDs exhibiting significant surface charge trapping, via a polaron (trapped charge/vibrational mode quasi-particle)-mediated transfer mechanism. For the latter type of coupling, trapped charges at the surface of the QDs must be present, whilst QDs with better surface quality will not exhibit this type of coupling. Note that both the polaron and the resonant energy transfer mechanisms are more significant than simple passive re-absorption of the PL by the DDT ligands in this IR range. We fabricated a plasmonic array with a pitch size of 1.2 μm and a first-order lattice resonance centered at 1775 ± 50 nm upon deposition of the 90-nm thick HgTe QD layer, denoted as Sample C in the Supporting Information, Fig. S2. In addition to the two-fold enhancement of the average spontaneous emission, matching the DDT vibration band to a first-order lattice resonance of the plasmonic array efficiently decreases the degree of non-radiative decay in this spectral range, providing a smooth, nearly Gaussian-shaped PL spectrum of the HgTe QDs (green curve in Fig. 4).

## Conclusions

We demonstrated the ability to boost and tailor the spontaneous near-IR to mid-IR emission of HgTe QDs via coupling to the localized and lattice SPPs of a fs-laser-printed plasmonic nanobump array. We achieved a five-fold enhancement of the PLQY of the HgTe QD layer on a plasmonic array, where different plasmonic resonances of the structure were appropriately matched to the absorption and PL bands of the attached QD emitters. Moreover, matching the first-order lattice resonance of the nanobump array with a vibration band of the stabilizing ligand of HgTe QDs (DDT) reduced the influence of this channel of non-radiative PL decay, allowing improvement of the shape of the emission spectrum. Importantly, the facile fabrication strategy used here provides a simple and reproducible way to produce high-quality plasmonic arrays with lateral dimensions that could be easily scaled up to squared centimeters, while their plasmonic response can be tuned via the geometrical shape of the isolated nanobumps and their spatial arrangement. Notably, in this study, we used a 90-nm thick layer of HgTe QDs to maintain a reasonable signal-to-noise ratio. For such a relatively thick layer (in plasmonic structure terms), the PL quenching effect is expected to be weak; however, it will be interesting to consider the interaction of a HgTe QD monolayer separated from the plasmonic array by a thin dielectric spacer (similar to the spacer used in the work by Chen et al.^35^) to achieve higher enhancement factors and stronger coupling, which will be a subject of our forthcoming studies. The realization of mid-IR to near-IR nanolasers based on the proposed approach can also be envisioned as a promising research direction.

## Materials and methods

### Synthesis of the HgTe QDs

The HgTe QDs used in this study were synthesized via a gaseous H_2_Te precursor by an aprotic solvent method, similar to that described in ref. ^22^. Initially, the QDs were grown in dimethylformamide solution using 2-furanmethanethiol as the ligand and mercury chloride as the metal salt precursor. After treating the solution with H_2_Te in a stream of argon carrier gas, the QDs thus formed were extracted from the solution, and the ligand was exchanged for dodecanethiol (DDT) to render the QDs soluble in tetrachloroethylene, which is suitable for IR absorption/emission spectroscopy in the IR range of interest. We utilized two types of pyramidal-shaped HgTe QDs with characteristic average sizes of 3.9 and 5.0 nm and emission maxima centered at 1.6 and 2.2 μm, respectively (Fig. 2b). QDs dissolved in tetrachloroethylene at a concentration of approximately 0.5 × 10^15^ QDs/ml were deposited above the plasmonic nanobump arrays using a drop-casting method to provide a uniform 90-nm thick layer of densely packed nanoparticles.

### Fabrication of IR-resonant plasmonic nanobump arrays

As a target for laser patterning, we used a 50-nm thick Au film deposited onto a glass substrate using a commercial *e*-beam evaporation system equipped with a calibrated quartz microbalance (Kurt J. Lesker Company, USA). Second-harmonic (515 nm) 200-fs laser pulses generated by a regeneratively amplified Yb:KGW laser system (Pharos, Light Conversion) were focused with a dry objective at a numerical aperture of 0.5 to yield a focal spot of approximately 1 μm in diameter. For large-scale array printing, Au film samples were arranged on a programmable nanopositioning platform providing a centimeter-scale traveling range at 50-nm movement precision. Such fabricated lattices had a size of 3 × 9 mm^2^ and contained approximately 10^8^ individual nanobumps that had a height of 600 ± 30 nm and a base diameter of 680 ± 30 nm and were arranged into rectangular-shaped arrays at variable pitch *p* for each array. The reproducibility of the fabrication process is shown in Fig. S6 of the Supporting Information, revealing no more than 4% FTIR amplitude difference and 30-nm precision in the resonance position along the whole fabricated array. The fabrication process of a single array takes approximately 30 min for single-beam scanning at a 200 kHz maximal pulse repetition rate and can be further speeded up via a beam multiplexing approach^49^.

### Characterization

The geometrical parameters of the isolated nanoantennas and their arrays were carefully analysed with a scanning electron microscope (SEM, Ultra 55+, Carl Zeiss). Cross-sectional cuts revealing the inner structure of the nanoantennas were produced using a focused Ga-ion beam (FIB) milling machine (Raith, IonLiNE). A 300-nm thick Ti over-layer was applied above the nanobumps prior to the FIB milling procedure to provide a clear interface SEM contact after ion beam slicing. The details of the milling procedure can be found elsewhere^46^. Reflection spectra of the bare and coated arrays were measured using a Fourier transform infrared (FTIR) microscope (Bruker, Hyperion 3000) coupled to an IR spectrometer (Bruker, Vertex 80 v). All measurements were made in reflection mode using a Cassegrain lens with 0.5 numerical aperture by averaging 1000 scans at a spectral resolution of 8 cm^−1^. All spectra were normalized to the signal measured from the smooth Au film surface. Absorption spectra were measured on a UV 3600 spectrometer (Shimadzu, Japan) in a 380–3000 nm spectral range. Steady-state PL spectra were measured on an FLS920P spectrometer system (Edinburgh Instruments, UK), and the scheme of the system is shown in Fig. S5. The PL spectra were measured using a liquid nitrogen-cooled photomultiplier tube covering the spectral range up to 1630 nm. PL spectra at longer wavelengths (up to 3500 nm) were measured with a nitrogen-cooled InSb detector. A 2-W 880-nm solid-state laser was used for PL excitation. Time-resolved PL measurements were performed by a time-correlated single photon counting technique at room temperature with a 200-ns decay window. A 62.8-ps pulse width pulsed diode laser with an emission wavelength of 670 nm was used for excitation during the decay measurements. The TEM images were captured by a field emission transmission electron microscope (FEI TecnaiF20) operating at 200 kV.

### FDTD simulations

3D FDTD simulations realized with a commercial software package (Lumerical Solutions, Inc.) were used to reveal the structure of the electromagnetic fields in the vicinity of the laser-pumped nanoantennas. Comprehensive SEM and FIB data were used to elaborate the exact 3D models of the nanoantenna used for these simulations. Simulations with an ultrafine 1-nm^3^ mesh were carried out considering linearly (*x*-axis) polarized plane-wave excitation from the top as well as periodic boundary conditions in both horizontal directions and perfectly matched layers as the boundary conditions limiting the vertical directions of the computation volume. To gain insight into the shape of the modeled nanobumps, a cross-sectional central cut was made using focused Ga^−^ ion beam milling. A side-view SEM image of this cut is shown in Fig. 1d. The dielectric constants of Au and SiO_2_ were taken from ref. ^54^. The scattering spectrum from the isolated nanobump of the same geometry has been simulated by considering the isolated nanobump excited from the top by a broadband linearly polarized total-field scattered-field source. In this case, the computational volume was limited by perfectly matched layers. The back-scattered spectrum was normalized over the excitation spectrum and averaged over the upper hemisphere of collection angles. The results of these simulations are presented in the Supporting Information, Fig. S2.

## Supplementary information

Supplementary Information for Tailoring spontaneous infrared emission of HgTe quantum dots with laser-printed plasmonic arrays.
