# Supplementary material for: Tailoring spontaneous infrared emission of HgTe quantum dots with laser-printed plasmonic arrays
Source: Light Sci Appl. 2020 Feb 4;9:16. doi: 10.1038/s41377-020-0247-6 (PMC7000696; doi:10.1038/s41377-020-0247-6)
Supplement: Supplementary file 1 — Supplementary Information for Tailoring spontaneous infrared emission of HgTe quantum dots with laser-printed plasmonic arrays. [file 41377_2020_247_MOESM1_ESM.pdf]

# Supporting information for

## Tailoring spontaneous infrared emission of HgTe quantum dots with laser-printed plasmonic arrays

A.A. Sergeev,[a] D.V. Pavlov,[a,b] A.A. Kuchmizhak,[a,b†] M.V. Lapine,[c] W.K. Yiu,[d] Y. Dong,[e,f] N. Ke,[f] S. Juodkazis,[g,h] N. Zhao,[f] S. V. Kershaw,[d] A. L. Rogach[d,†]

[a]Institute of Automation and Control Processes, Far Eastern Branch, Russian Academy of Sciences, Vladivostok 690041, Russia

[b]Far Eastern Federal University, Vladivostok 690090, Russia

[c]University of Technology Sydney, NSW 2007, Sydney, Australia

[d]Department of Materials Science and Engineering, and Centre for Functional Photonics (CFP), City University of Hong Kong, Kowloon, Hong Kong S.A.R. , China

[e]Engineering Research Center of Nano-Geomaterials of Ministry of Education, Faculty of Material Science and Chemistry, China University of Geosciences, Wuhan 430074, China

[f]Department of Electronic Engineering, The Chinese University of Hong Kong, Shatin, New Territories, Hong Kong S. A. R. , China

[g]Swinburne University of Technology, John St., Hawthorn 3122, Victoria, Australia

[h]Melbourne Centre for Nanofabrication, ANFF, 151 Wellington Road, Clayton, VIC3168, Australia

[†]corresponding author: alex.iacp.dvo@mail.ru (A.A.K), andrey.rogach@cityu.edu.hk (A.L.R.)

## Tuning first-order lattice resonance through the nanobump geometry and array pitch.

In this section we demonstrate tunability of the first-order lattice plasmon resonance (FLPR) of the nanobump array via tailoring the nanobump geometry as well as via array pitch dimensions *p*. Two representative series of FTIR reflection spectra demonstrate red-shift of the FLPR spectral position caused by either an increase of the geometric size of the nanobumps tailored by increasing the applied pulse energy in the process of their laser printing (left-side column) as well as by increasing the array pitch *p* for the fixed size of the nanobumps in the array (fixed pulse energy applied; right-side column). In the first case, the red shift of the FLPR is caused by increased “effective” array period (or plasmon running distance) accumulated on the curved surface of the nanobumps.

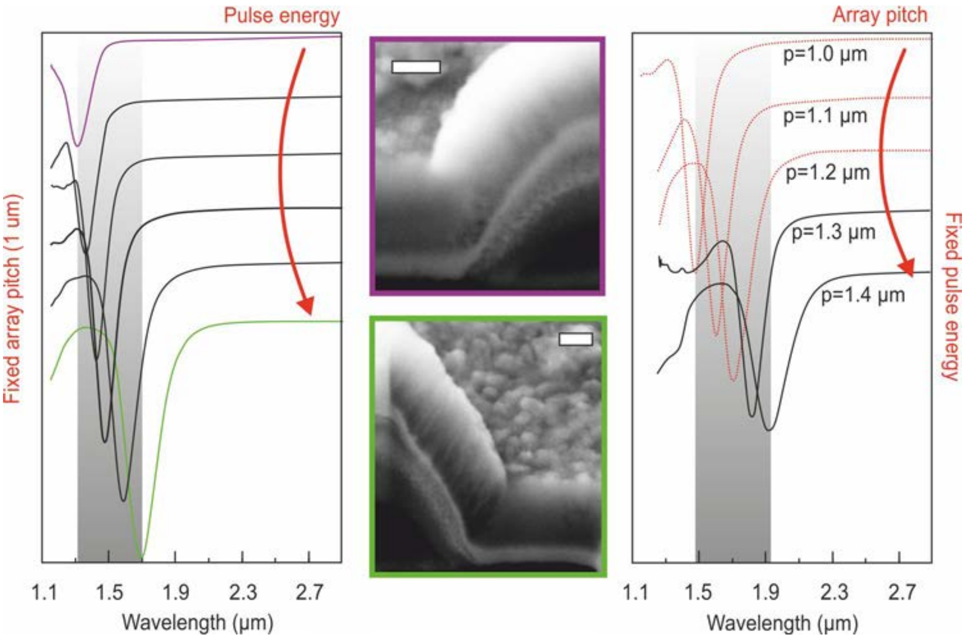


Figure S1: Series of FTIR reflection spectra showing the ability to tailor a spectral position of the FLPR via either a nanobump geometry tuned by applied pulse energy (left-side column) or an array pitch at fixed nanobump size (right column). FTIR spectra are vertically shifted for better representation. Shaded areas on both series indicate the FLPR tuning range. Two inset SEM images show the FIB cross-section central cut through the nanobumps produced at the lowest (purple) and the highest (green) pulse energy shown on the left-side column. Three spectra on the right-side column shown as red dotted curves correspond to arrays used in the manuscript as samples A, B and C.

To further illustrate this point, we provide two SEM images showing the FIB cross-section central cut through the nanobumps produced at the lowest (purple curve) and the highest (green curve) pulse energy. Careful analysis of the geometrical size of both structures indicate that the smallest nanobumps increase the actual array pitch by ≈ 300 nm, while the largest ones - by 700 nm, which is in satisfactory agreement with the experimentally observed spectral shift of the FLPR. From the other hand, for the fixed size of the nanobumps, an increase of the array pitch also results in a scalable red shift of the FLPR position. The provided results also indicate that the efficiency of coupling to the surface plasmon waves depends on both the nanobump geometry and their spacing. Indeed, smaller or too separated nanobumps provide weaker coupling efficiency. Notably, maximal coupling efficiency, which can be assessed by the FLPR amplitude on the FTIR spectrum, was about 40% for large-scale nanobump arrays produced using direct laser printing.

## Spectral position of the plasmonic lattice resonances

As it was shown in refs. [1, 2], nanobump arrays support first-order lattice plasmon resonances at a wavelength determined by the plasmon running length (or effective array period). The latter comprises the slopes of the nanobump sidewalls, plus the remaining flat surface between the structures, and so it is greater than the array period (see inset in Fig. 1b). At normal incidence, when the plasmon wavelength

$$\lambda_{SP} = \lambda_0 \operatorname{Re}\sqrt{\frac{1}{\varepsilon_m}+\frac{1}{\varepsilon_s}} \qquad (1)$$

(where $\lambda_0$ is the wavelength in free space, $\varepsilon_m$ and $\varepsilon_s$ are the complex permittivities of the metal layer and of the superstrate), is equal to the effective period, the plasmons interfere in phase and a resonance is observed.

Fig. S3 shows the theoretically calculated spectral position of the first- and the second-order lattice resonances (red and green lines, respectively) as a function of the effective period of the Au nanobump array. Spectral positions of the first-order lattice resonance of three samples A, B and C used in this work are provided, as well. Note that the resonances presented here, correspond to the bare arrays; with a HgTe QD layer added, the resonance is red-shifted by approximately 90 nm through the change of the plasmon wavelength with the added material.

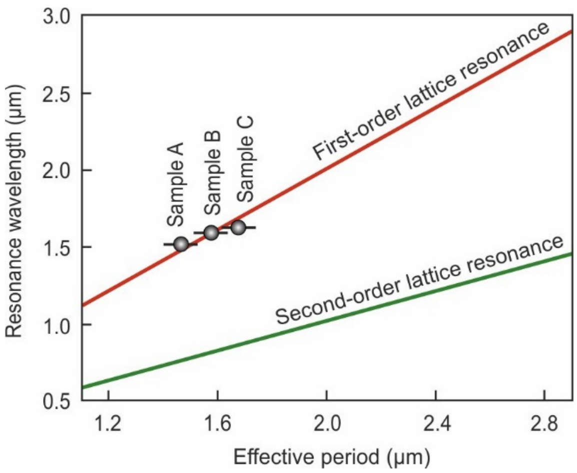


Figure S2: Calculated spectral positions of the first- and the second-order lattice resonances (red and green lines, respectively) as a function of the effective period of the Au nanobump array. Experimental data from three samples A, B and C used in this work are shown as black circles.

The second harmonic of this resonance corresponds to the wavelength when the effective period of the nanobump array equals to double the plasmon wavelength. This resonance can be tailored by geometry and arrangement of the nanobumps in a similar way to fit the pump laser wavelength. This could allow for more efficient excitation, albeit this resonance has much weaker amplitude due to a stronger plasmon dissipation in the visible spectral range.

## Localized surface plasmon resonances of isolated nanobumps

We performed 3D FDTD calculations to reveal the spectral range where the isolated nanobumps can contribute to enhanced electromagnetic fields via excitation of LSPRs. For these calculations, we considered an isolated nanobump having the geometry shown in Fig. 1b, which was excited from the top by a broadband linearly polarized total-field scattered-field source. Computational volume was limited by perfectly matched layers. The back-scattered spectrum normalized over the excitation spectrum and averaged over the upper hemisphere collection angles is shown in Fig. S3 revealing multiple LSPRs at wavelengths below 1100 nm. This spectrum is also shown in an inverted view in Fig. 1c of the main manuscript indicating good correlation with the experimental results and explaining the broadband dip in reflection in the visible and near-IR spectral ranges.

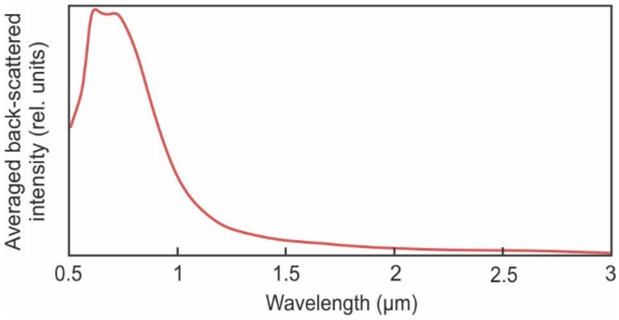


Figure S3: Back-scattered spectrum of the isolated Au nanobump normalized over the excitation spectrum and averaged over the upper hemisphere collection angles.

## Calibration of the spectral shift of the first-order lattice resonance versus the thickness of the capping dielectric layer

To assess the spectral shift of the main lattice band of the Au nanobump array (centered at $\lambda_0$) which is caused by the deposition of the HgTe QD layer, we have performed a series of calibration experiments using an amorphous silicon (α-Si) film, deposited above several similar plasmonic nanobump arrays using a commercial e-beam evaporation system equipped with a calibrated quartz microbalance. This material has averaged bulk refractive indices of $n_{\alpha-Si} \approx 3.47$ in the near-IR spectral range [3], which is expected to be close to the bulk refractive index of the HgTe QDs, according to the available experimental data. The experimentally measured relative spectral shift of the main lattice band $\Delta\lambda/\lambda_0$ as a function of the thickness of α-Si capping layer, *d*, is presented in Fig. S4.

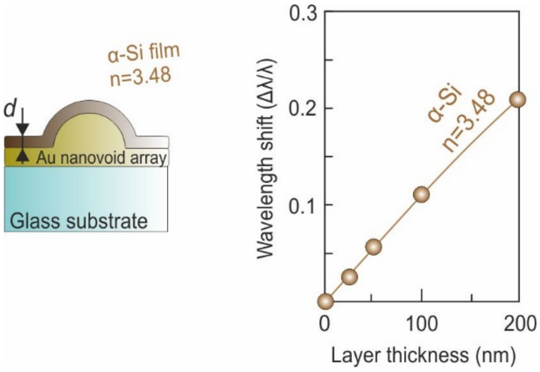


Figure S4: (left) Schematic illustration of the considered sample geometry and (right) relative spectral shift $\Delta\lambda/\lambda_0$ of the first-order lattice resonance of the Au nanobump array versus the thickness, *d*, of α-Si thin films deposited on the top.

## NIR PL setup used in this study

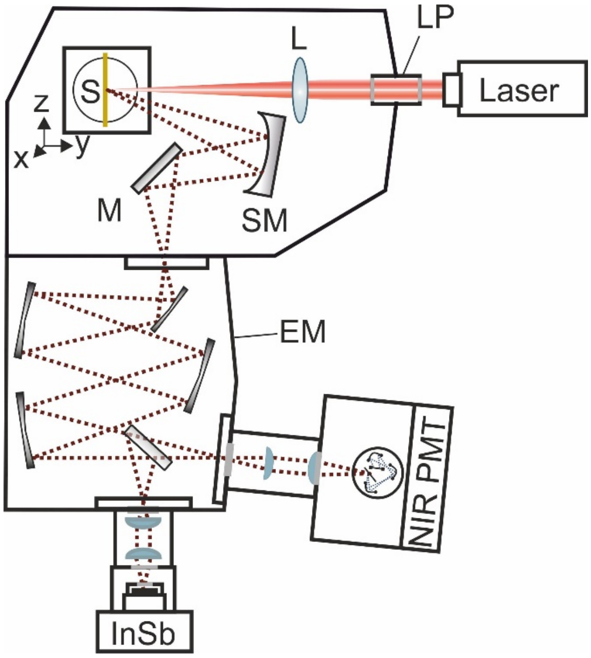


Fig. S5. The schematic drawing of FLS920P spectrometer system used in this study. “Laser” – 2 W, 880 nm solid state laser for steady-state measurements or 670 nm, 62.8 ps pulse width pulsed laser for time-resolved measurements; “LP” – custom manufactured laser port for PL excitation by external laser; “L” – focusing laser lens, f = 150 mm; “S” – sample on XYZ-translation and rotation stage for its precise adjusting with collection optics; “SM” – spherical mirror for emission collection; “M” – plane mirror; “EM” – emission monochromator; NIR PMT – nitrogen-cooled photomultiplier tube used for time-resolved measurements (spectral range 550-1630 nm); InSb - nitrogen-cooled solid state detector used for PL steady state measurements (spectral range 1200-4500 nm).

**The reproducibility of fabrication technique and corresponding PL enhancement**

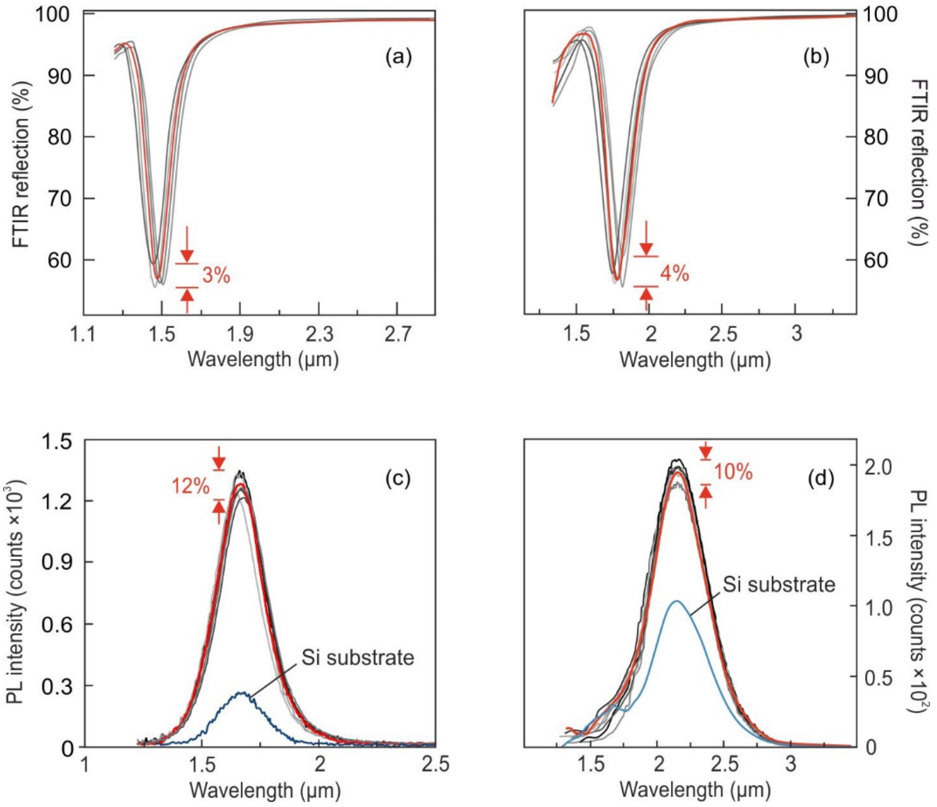


Fig. S6. A series of FTIR spectra of as-fabricated plasmonic nanobump arrays (a, b) and corresponding emission spectra of the HgTe QD layers deposited on plasmonic arrays and reference Si substrates (c, d) measured at a range of different points on the fabricated structures. Figures (a, c) and (b, d) correspond to Sample A and Sample C, respectively.

## References


1. D Pavlov, S Syubaev, A Kuchmizhak, S Gurbatov, O Vitrik, E Modin, S Kudryashov, X Wang, S Juodkazis, M Lapine. Direct laser printing of tunable IR resonant nanoantenna arrays. *Applied Surface Science* 469, 514 (2019)

2. D Pavlov, S Gurbatov, SI Kudryashov, PA Danilov, AP Porfirev, SN Khonina, OB Vitrik, SA Kulinich, M Lapine, AA Kuchmizhak.10-million-elements-per-second printing of infrared-resonant plasmonic arrays by multiplexed laser pulses. *Optics letters* 44 (2), 283 (2019).

3. Pierce, D. & Spicer, W.E. Electronic structure of amorphous Si from photoemission and optical studies. *Phys. Rev. B.* 5, 3017 (1972).
